# Supplementary material for: Reactivity of He with ionic compounds under high pressure
Source: Nat Commun. 2018 Mar 5;9:951. doi: 10.1038/s41467-018-03284-y (PMC5838161; doi:10.1038/s41467-018-03284-y)
Supplement: Supplementary file 1 — Supplementary Information [file 41467_2018_3284_MOESM1_ESM.pdf]

Supplementary Information

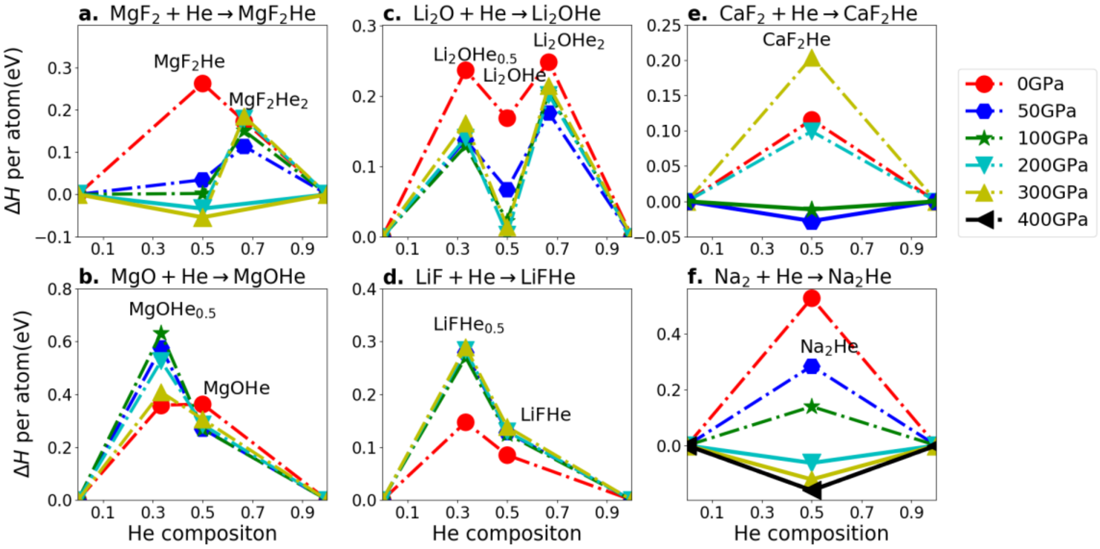

Supplementary Figure 1. The enthalpies of reactions. Calculations between Helium and **(a)** MgF_2, **(b)** MgO, **(c)** Li_2O, **(d)** LiF, **(e)** CaF_2 ad **(f)** Na_2, plotted as function of Helium content. The convex hulls connecting thermodynamically stable compositions for each pressure are drawn as solid lines. Unstable compounds are connected with dashed lines. The pressure range in **(a)-(e)** is 0 to 300GPa, and in **(f)** it is 0 to 400GPa. The x-axis denotes $x/(x+y)$ for the compound $(A_mB_n)_yHe_x$.

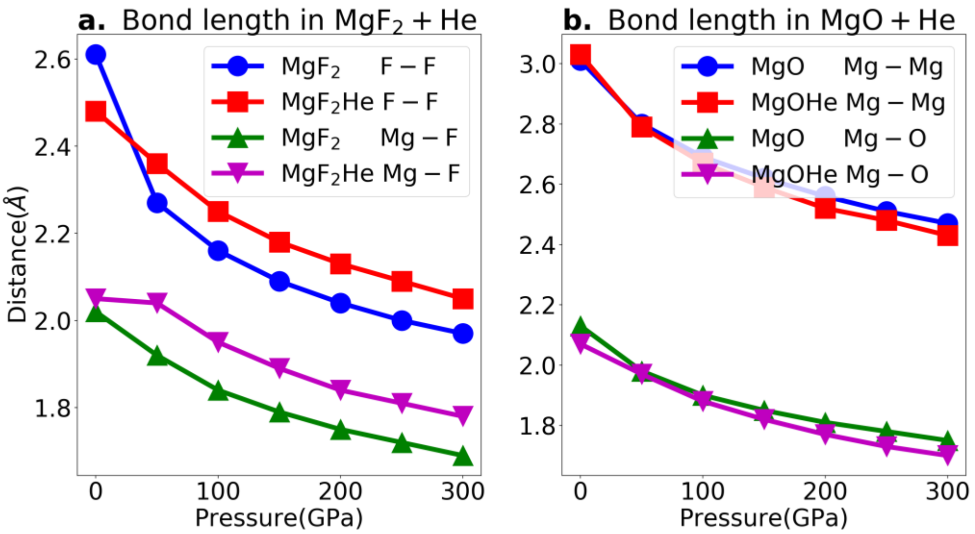

Supplementary Figure 2. Ionic distance as function of pressure. (**a**) The shortest ionic separations with and without He inclusions, including F-F and Mg-F distances in MgF_2 and MgF_2He; (**b**) Mg-Mg and Mg-O distances in MgO and MgOHe.

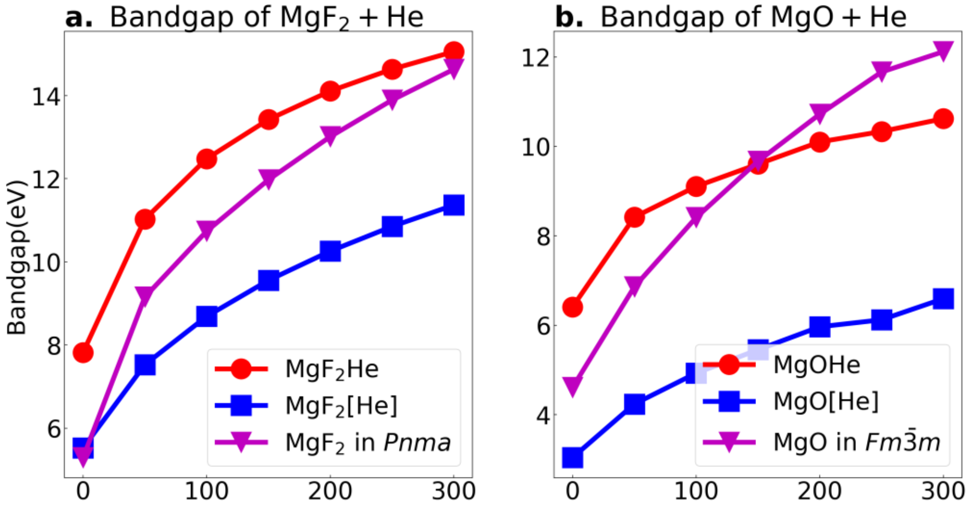

Supplementary Figure 3. Band gaps as function in terms of pressure. (**a**) Band gaps as a function of pressure for MgF_2 in the Pnma structure, MgF_2He in the Full-Heusler structure, and $MgF_2[He]$; (**b**) Band gaps as a function of pressure for MgO in the Rocksalt structure, MgOHe and MgO[He];

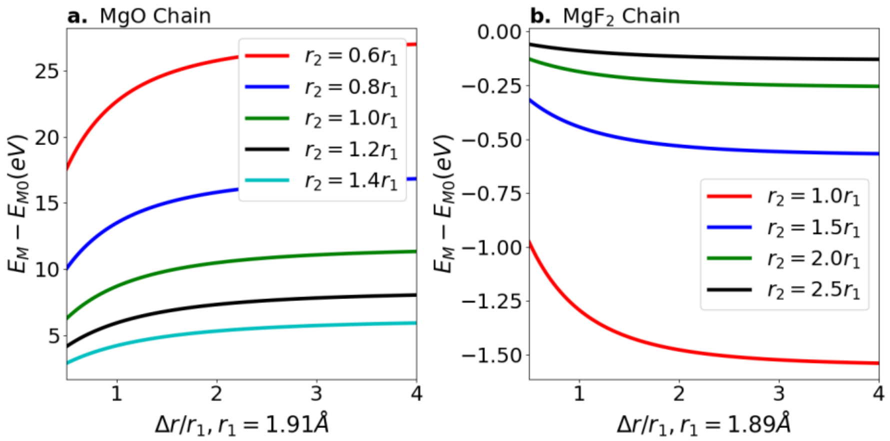

Supplementary Figure 4. Madelung energy change in 1D chain. The change of the Madelung energy before (E_{M0}) and after (E_M) the insertion of He atoms as a function of the "size" of the inserted dummy atom, for particular **(a)** AB and **(b)** A_2B compounds, respectively. For AB, r_1 is the distance from A to B (e.g. blue then red in Fig. 5 in the main text) while $r_2 = \lambda r_1$ is the one from B to A (e.g. red then blue). The size of the dummy atom is Δr. For A_2B, r_1 is the distance between ion A and B (blue and red in Fig. 5 in the main text), while $r_2 = \lambda r_1$ is the one between 2 adjacent A ions (2 neighboring red circles).

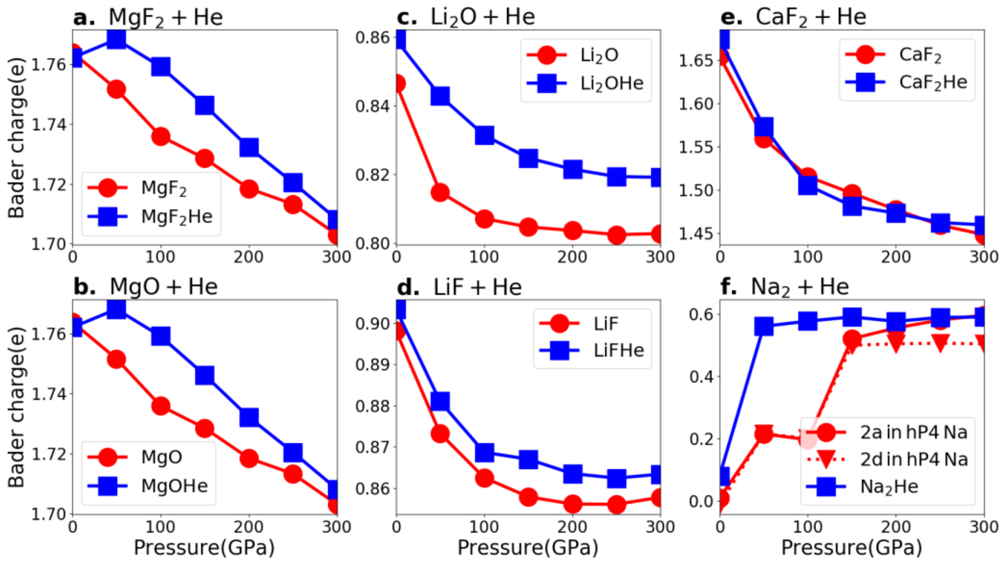

Supplementary Figure 5. Bader charges on cations as a function of pressure. The 2a and 2d in (f) stand for the Na Wyckoff position in the *hP*4 structure.

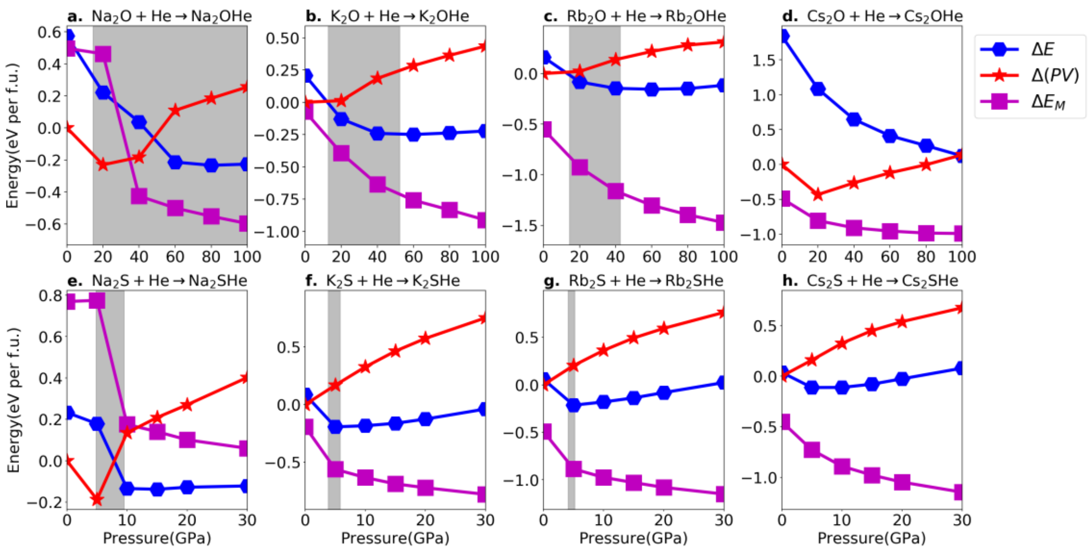

Supplementary Figure 6. Energy differences as function of pressure. Relative changes in PV work and internal energy E for the given He-inclusion reaction, as well as numerically determined Madelung energies (E_{M}) of the He-inclusion compounds as a function of pressure. Eight alkali metal compounds are chosen, including the oxides and sulfides of Na, K, Rb and Cs. Shaded areas present the stable pressure interval.

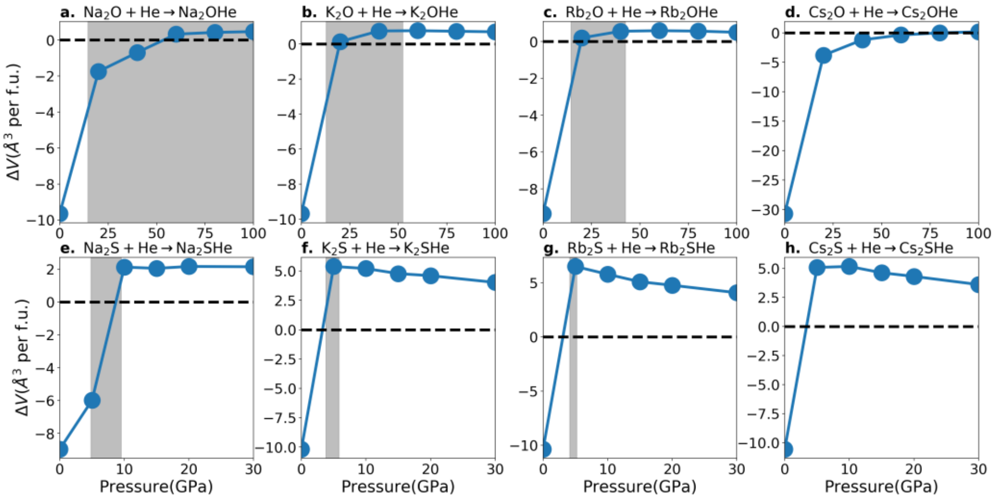

Supplementary Figure 7. The changes of the volume as function of pressure for the He insertion reactions. The dashed lines refer to the volume of the unreacted ionic compound and elemental Helium. Eight alkali metal compounds are chosen, including the oxides and sulfides of Na, K, Rb and Cs. Shaded areas present the stable pressure interval.

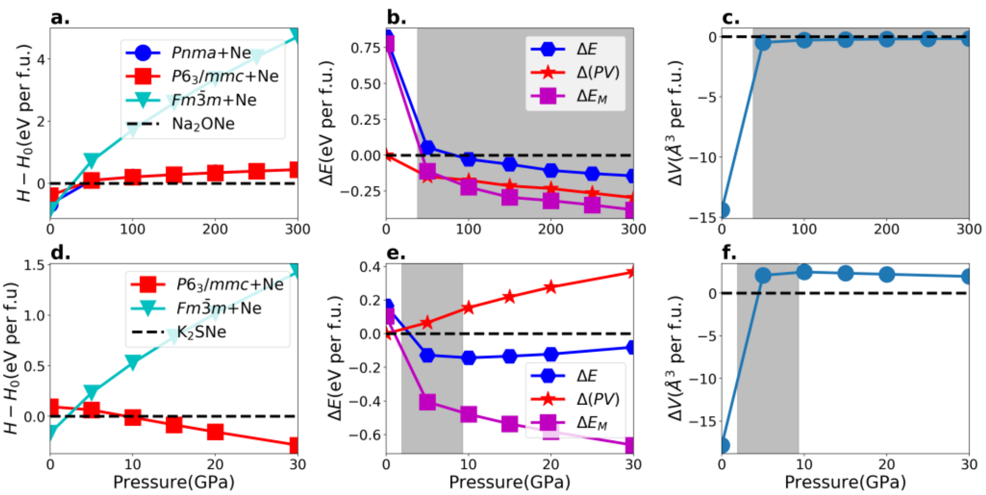

Supplementary Figure 8. The stability and energy and volume analysis of Ne inserted ionic compounds. **(a)** and **(d),** the enthalpies as function of pressure for Na_2O (and K_2S) compounds and Ne solid, in comparison with the enthalpy of Na_2ONe (and K_2SNe). **(b)** and **(e),** relative changes in *PV* work and internal energy *E* for the given He-inclusion reaction, as well as numerically determined Madelung energies (E_M) of the He-inclusion compounds as a function of pressure. **(c)** and **(f),** the changes of the volume as a function of pressure for the He insertion reactions. The dashed lines refer to the volume of the unreacted ionic compounds and elemental Helium. Shaded areas present the stable pressure interval.

Supplementary Table 1. Ion distances. The nearest neighbor interatomic distances of the studied ionic compounds and their He inserted products under pressure (Å). E stands for the quasi-atom in the electrides containing Na.

	Pressure(GPa)	0	50	100	150	200	250	300	350	400
Li_2O	Li-Li	2.33	2.08	1.97	1.88	1.80	1.75	1.71		
	O-O	3.29	2.74	2.59	2.49	2.40	2.34	2.30		
	Li-O	2.01	1.76	1.67	1.62	1.59	1.56	1.54		
Li_2OHe	Li-Li	2.21	2.18	2.07	1.99	1.94	1.90	1.86		
	O-O	3.12	3.08	2.92	2.82	2.74	2.68	2.63		
	Li-O	1.93	1.89	1.79	1.73	1.68	1.64	1.61		
LiF	Li-Li	2.89	2.57	2.44	2.36	2.30	2.25	2.21		
	F-F	2.89	2.57	2.44	2.36	2.30	2.25	2.21		
	Li-F	2.04	1.81	1.72	1.67	1.62	1.59	1.56		
LiFHe	Li-Li	2.73	2.60	2.46	2.37	2.31	2.26	2.22		
	F-F	2.73	2.60	2.46	2.37	2.31	2.26	2.22		
	Li-F	1.93	1.83	1.75	1.68	1.63	1.60	1.57		
MgF_2	Mg-Mg	3.10	2.98	2.83	2.73	2.65	2.60	2.55		
	F-F	2.61	2.27	2.16	2.09	2.04	2.00	1.97		
	Mg-F	2.02	1.92	1.84	1.79	1.75	1.72	1.69		
MgF_2He	Mg-Mg	3.14	3.33	3.18	3.08	3.01	2.95	2.90		
	F-F	2.48	2.36	2.25	2.18	2.13	2.09	2.05		
	Mg-F	2.05	2.04	1.95	1.89	1.84	1.81	1.78		
MgO	Mg-Mg	3.01	2.80	2.69	2.62	2.56	2.51	2.47		
	O-O	3.01	2.80	2.69	2.62	2.56	2.51	2.47		
	Mg-O	2.13	1.98	1.90	1.85	1.81	1.78	1.75		
MgOHe	Mg-Mg	3.03	2.79	2.67	2.59	2.52	2.48	2.43		
	O-O	3.03	2.79	2.67	2.59	2.52	2.48	2.43		
	Mg-O	2.07	1.97	1.88	1.82	1.77	1.73	1.70		
CaF_2	Ca-Ca	3.89	3.12	2.89	2.90	2.83	2.77	2.73		
	F-F	2.75	2.44	2.27	2.24	2.19	2.15	2.11		
	Ca-F	2.38	2.10	2.04	1.97	1.93	1.89	1.86		
CaF_2He	Ca-Ca	3.98	3.57	3.40	3.29	3.21	3.15	3.09		
	F-F	2.81	2.52	2.40	2.33	2.27	2.22	2.19		
	Ca-F	2.43	2.18	2.08	2.01	1.97	1.93	1.89		
Na	Na-Na	3.69	2.66	2.48	2.07	2.00	1.94	1.90	1.86	1.78
	E-E	3.69	2.66	2.48	2.93	2.76	2.63	2.55	2.49	2.40
	Na-E	2.17	1.56	1.43	1.69	1.67	1.65	1.62	1.60	1.52
Na_2He	Na-Na	3.27	2.48	2.28	2.16	2.09	2.03	1.98	1.94	1.91
	E-E	4.62	3.51	3.22	3.06	2.95	2.87	2.80	2.75	2.70
	Na-E	2.83	2.15	1.97	1.88	1.81	1.76	1.72	1.68	1.65

Supplementary Note 1. Convex Hull

To compare the stability of the products, we calculated the convex hull of formation enthalpy. For the chemical reaction $\mathrm{A}_m\mathrm{B}_n + x * \mathrm{He} \rightleftharpoons \mathrm{A}_m\mathrm{B}_n\mathrm{He}_x$, the formation enthalpy can be described as below:

$$\Delta H = \frac{1}{m+n+\mathrm{x}}[H(\mathrm{A}_m\mathrm{B}_n\mathrm{He}_x) - H(\mathrm{A}_m\mathrm{B}_n) - x * H(\mathrm{He})] \quad (1)$$

in which ΔH is the formation enthalpy per atom in the predicted product and each H on the right hand side is the enthalpy of the corresponding compound, evaluated with the crystal structure of the lowest enthalpy at the given pressure conditions. If plotted against the Helium concentration calculated via. $\frac{x}{x+y}$ for $(\mathrm{A}_m\mathrm{B}_n)_y\mathrm{He}_x$, the convex hull of the relative enthalpies ΔH designates all compounds that are stable against decomposition (those whose ΔH lies on the hull).

Supplementary Figure 1 shows the calculated convex hulls for the ionic compounds studied in the main text, as well as the convex hull for the Na_2He compound that was found to be stable under high pressure by Dong *et al.*[1] Generally speaking, the results support our hypothesis. They show that when the cation:anion ratio is 1:1, the insertion of He will either not stabilize the structure at all or only marginally. In contrast, when the cation:anion ratio is not 1:1, the insertion of He has a significant stabilising effect and often stabilizes the $(\mathrm{A}_m\mathrm{B}_n)$He compounds within a particular pressure range.

Supplementary Note 2. Effects of He insertion to ionic compound structure

The effect of the insertion of He in ionic compounds can be analysed through the change of their structure, especially the interatomic distances. For example, the insertion of He in Na, which is actually Na_2E, changes both the structure and the interatomic distances. At 200 GPa, the stable structure for pure Na is the insulating electride *hP*4 phase (seen as the Ni_2In structure type, with Na (E) on the Ni (In) site, respectively),[1] whereas Na_2He has the fluorite structure (CaF_2). If we compare the interatomic distances, an important difference between the two structures is that the Na-Na distances are larger in the fluorite structure; which would suggest reduced repulsive cation-cation interactions in

the He-inclusion compound. At 200GPa, for each Na atom in Na_2He, there are 26 Na atoms within 4 Å, including 6 nearest neighbors at 2.16 Å, 12 second nearest neighbors at 3.06 Å, and 8 third nearest neighbors at 3.75 Å. For comparison, in the *hP*4 structure[2] at the same pressure there are also 26 Na atoms within 4 Å, which can be classified into three groups. The first group contains 6 nearest neighbors at 2.07 Å, which is 4.2% shorter than in Na_2He. The second group contains neighbors at distances of 2.45 Å (2 atoms), 2.90 Å (6 atoms) and 2.97 Å (6 atoms). Compared to the second nearest neighbor distances in Na_2He these values are 20/5.2/2.94% shorter. The third group consists of six atoms at 3.57 Å, which is 4.8% shorter than the third nearest neighbor distance in Na_2He.

Applying the same idea, we can compare the distances between ions in MgF_2 vs MgF_2He, and in MgO vs MgOHe. In Supplementary Figure 2a, we show the nearest-neighbor F-F and F-Mg separations for MgF_2 and MgF_2He. At high pressure, the shortest F-F separations in the MgF_2He structure are larger than in MgF_2, which suggests that the anion-anion repulsion is reduced in MgF_2He. Note that the Mg-F distance increases from MgF_2 to MgF_2He, but this increment is small. In MgO and MgOHe, which are shown in Supplementary Figure 2b, the Mg-O and Mg-Mg distances show almost no difference upon inclusion of He, which suggests less of a Coulomb stabilisation effect.

Of course, electrostatic interactions are of long-range character, and examining the local environment of the various ions in these systems is suggestive but hardly complete. The appropriate measure of the overall balance between Coulomb attraction and repulsion is the Madelung energy, and we will show in the section of *The driving force of He insertion* in the main text, for instance, the Madelung energy of MgF_2 is lowered at high pressure by introducing He atoms, whereas this is not the case for MgO.

Supplementary Note 3. Effects of He insertion to band gaps

Although He atoms do not form local chemical bonds with the neighboring atoms, their insertion into the crystal lattice can change the states of the other electrons. This can be well seen from the change of the electronic band gaps upon He insertion. In Supplementary Figure 3, we plot the band gaps of MgF_2, MgO and their He inserted compounds as a function of pressure. For

comparison, we also calculate the band gaps of hypothetical compounds created by removing all the He atoms from the He insertion compounds. All gaps increase with pressure. For both MgF_2[He] and MgO[He], the band gaps drop by about 4 eV after removing He atoms and evolve with increasing pressure almost in parallel with their respective insertion compound. The presence of the He atoms perturbs the ions' electron states through Pauli repulsion, thereby increasing their kinetic energies. This is felt more strongly by the conduction Mg-s states than the localised valence F-p and O-p states, and therefore increases the band gap. On the other hand, the band gaps of ionic compounds without He insertion increase faster than those of the He insertion compounds. This is consistent with the larger volume reduction of MgF_2 and MgO under increasing pressure. More details will be discussed in the next section. Although these results reveal significant effects of He insertion on electron states in the ionic compounds, these reciprocal space analyses can not single out the major driving force of forming the stable compounds. The maximum hardness principle could suggest that MgF_2He (with its larger gap) is more stable than MgF_2, certainly more so than MgOHe compared to MgO, but the agreement with the reaction enthalpies (see Fig. 1 in the main text) is tenuous. Instead, as we show in the manuscript, the stabilisation mechanism can be more clearly revealed by analyzing the electrostatic interactions in real space.

Supplementary Note 4. The one-dimensional model

The 1D ionic chain model can be solved analytically. For an AB compound, there are two neighboring distances, d(B-A) (from blue circle to red circle, following the convention from Fig. 5 in the main text) and d(A-B) (from red circle to blue circle). We set the distance d(B-A)=r_1, and the distance d(A-B)=r_2=λr_1. The corresponding Madelung energy can be obtained as:

$$E_M = \frac{e^2}{4\pi\epsilon_0 r_1}[-4 + \sum_{i=1}^{\infty} 4(\frac{2}{i(\lambda+1)} + \frac{-1}{i(\lambda+1)+1} + \frac{-1}{i(\lambda+1)-1})] \quad (2)$$

The insertion of a He atom in the 1D lattice of ions will only change d(A-B); which manifests as an increase of λ in the above formula. Half of the A-B pairs will thus be separated by He atoms while the other half will remain adjacent. In Supplementary Figure 4a we plot the Madelung energy as function of the

change of d(A-B) caused by the insertion. A similar formula can also be obtained for He inserted in A_2B types of ionic compounds. The major difference there is that He will always be inserted between an A-A pair. The corresponding Madelung energy change is plotted in Supplementary Figure 4b for A_2B types of compounds. As shown in Supplementary Figure 4, it is clear that the insertion of He in A_2B type compounds will *lower* the Madelung energy whereas the insertion in AB type compound will *raise* the Madelung energy. The magnitude of this change in energy strongly depends on the size of the inserted neutral atom, Δr, as well as on the original bond lengths d(A-A) and d(A-B).

Of course such a simplified 1D model is very different from real 3D materials. The change of the Madelung energy in 3D ionic compounds, determined numerically, is discussed in the main text. However, it is interesting to note that our 1D chain models correspond to arrangements of lines of atoms that are found in the actual structures of the AB and A_2B types of compounds. For example, for MgF_2 in the CaF_2 structure, the atom arrangement along the cubic [111] direction is identical to the 1D AB_2 model (Fig. 2e in the main text). The corresponding r is 1.89Å at 100 GPa. Similarly, the line of atoms along the [100] direction in the MgO rocksalt structure is identical to the 1D AB model (Fig. 2f in the main text).

Supplementary Note 5. The energy analysis of He reacting with alkali chalcogenides

The formation of He inserted alkali metal oxides and sulfides have been studied.[3] It was found that He can form stable compounds with most of the alkali metal oxides and sulfides under high pressure. However, when pressure increases, most of the He inserted compounds become unstable. The formation and instability of these compounds can be explained by our theory.

First, we repeated the work of Sun *et. al.* [3] and confirmed that most of the alkali metal compounds can react with He under high pressure. The enthalpy-vs-pressure plots are essentially the same as Fig. 2 in their paper.[3] Furthermore, we calculated the changes of the PV term, the internal energy and the Madelung energy for all eight compounds. These results show that the

Madelung energy greatly reduces while inserting He into the ionic compounds and becomes more negative with increasing pressure. It again clearly reveals that the reduction of the Madelung energy is the driving force for the insertion of He.

Following the study of Sun *et al.*[3] for the reactions of He with alkali chalcogenides, we analyze the components of their enthalpies as functions of pressure. As shown in that work, Li_2O, Li_2S, Na_2S and Cs_2O do not react with He. In contrast, Na_2O, K_2O, K_2S, Rb_2O, Rb_2S, and Cs_2S may form stable compounds with He within a certain pressure range. Beyond that pressure range, the unreacted ionic compounds in their high-pressure structures (usually *Pnma*) and elementary He become the more stable form. By analyzing the enthalpy components and calculating the Madelung energies, we find that the Madelung energy in these compounds decreases with He insertion, *i.e.* the driving force for He reactivity is always present. It is the counterbalance of other factors that destabilizes the He insertion compounds. These factors include the increase of the volume and the increase of the internal energy. For many of these compounds, such as K_2O, Rb_2O, K_2S, Rb_2S and Cs_2S, the internal energies decrease first with increasing pressure and beyond a certain point become larger. This trend of internal energy change strongly correlates with the change of volume for He insertion. At similar pressures, the total volume of A_2B/AB_2 compounds and elemental He becomes smaller than the He inserted ionic compound, *i.e.* $\Delta V>0$ in Supplementary Figure 7. Similar to CaF_2, the above trends of ΔE and ΔV under increasing pressure are caused by the occupation of the *d* orbitals in the alkali atoms.

Supplementary Note 6. Stability of Ne inserted ionic compounds

The proposed driving force and the factors opposing He insertion in ionic compounds may go beyond He and be applicable to other NG elements. We tested the stability of Ne inserted ionic compounds including Na_2O and K_2S. The insertion of Neon in K_2S has been studied in a previous work.[3] Three structures are chosen for the ionic compounds including *Pnma*, $P6_3/mmc$ and $Fm\bar{3}m$. The full-Heusler structure is used for the Ne inserted compounds. For K_2S and K_2SNe, these structures are the same as used in the work of Sun *et. al.*[3] For Na_2O and Na_2ONe, we thoroughly searched the structures at 100, 200 and 300 GPa. The

structure search confirms that the most stable structure for Ne inserted compound is the full-Heusler structure, and the *Pnma*, $P6_3/mmc$ and Fm-3m structures are the lowest energy structures.

As shown by our calculations, Ne can form stable compound with Na_2O at a pressure higher than 50 GPa. The energy analyses show that the reduction of the Madelung energy is the major driving force. In contrast to He insertion, the PV term decreases continuously throughout the pressure range.

Comparing to Na_2O, Neon can form a stable compound with K_2S at a pressure higher than 2 GPa. However, the Ne inserted compound becomes unstable at higher pressure (> 10 GPa). This phenomenon is very similar to the insertion of He in the K_2S compound. It is caused by the occupation of K 3d orbitals.

Supplementary References

1. Dong X., *et al.* A Stable Compound of Helium and Sodium at High Pressure. *Nat. Chem.* **9**, 440-445 (2017).

2. Ma Y., *et al.* Transparent Dense Sodium. *Nature* **458**, 182 (2009).

3. Sun J., *et al.* Formation of Noble Gas Compounds with Alkali Oxides and Sulfides under Pressure. *arXiv:1409.2227*, 5 (2014).
